# A guide to applying the Good Publication Practice 3 guidelines in the Asia-Pacific region

**DOI:** 10.1186/s41073-019-0079-1

**Published:** 2019-10-02

**Authors:** Blair R. Hesp, Katsuhisa Arai, Magdalene Y. S. Chu, Stefanie Chuah, Jose Miguel B. Curameng, Sandeep Kamat, Zhigang Ma, Andrew Sakko, Hazel Fernandez

**Affiliations:** 1 Kainic Medical Communications Ltd, 104 Bond Street, Dunedin, 9016 New Zealand; 2 Proscribe Medical Affairs, Envision Pharma Group, Tokyo, Japan; 3 MIMS (Hong Kong) Ltd, Causeway Bay, Hong Kong; 4 AMICULUM (Singapore) Pte Ltd, Singapore, Singapore; 5 Cactus Communications Pvt Ltd, Mumbai, India; 6 AstraZeneca China, Shanghai, China; 7 Syneos Health, Gordon, NSW Australia; 8 AstraZeneca Australia, Macquarie Park, NSW Australia

**Keywords:** Asia-Pacific, Authorship, Conflict of interest, Disclosures, Ethics, Good Publication Practice, Manuscript development

## Abstract

Numerous recommendations and guidelines aim to improve the quality, timeliness and transparency of medical publications. However, these guidelines use ambiguous language that can be challenging to interpret, particularly for speakers of English as a second language. Cultural expectations within the Asia-Pacific region raise additional challenges and several studies have suggested that awareness and application of ethical publication practices in the Asia-Pacific region is relatively low compared with other regions. However, guidance on applying ethical publication practice guidelines in the Asia-Pacific region is lacking. This commentary aims to improve publication practices in the Asia-Pacific region by providing guidance on applying the 10 principles of the Good Publication Practice 3 (GPP3) guidelines and the International Committee of Medical Journal Editors (ICMJE) criteria for authorship. Recommendations are provided for encore presentations, applying the ICMJE authorship criteria in the context of regional cultural expectations, and the role of study sponsors and professional medical writers. Ongoing barriers to compliance with guidelines are also highlighted, and additional guidance is provided to support authors submitting manuscripts for publication. The roles of regional journals, regulatory authorities and professional bodies in improving practices are also discussed.

**Electronic supplementary material:**

The online version of this article (10.1186/s41073-019-0079-1) contains supplementary material, which is available to authorized users.

## Introduction

Numerous recommendations and guidelines have been developed to improve the quality, timeliness and transparency of publishing medical data (see Additional file 1 for a list of relevant recommendations and guidelines). These include the International Committee of Medical Journal Editors (ICMJE) recommendations (Table 1) [1], which provide guidance on all aspects of medical research published in peer-reviewed journals, and the Good Publication Practice 3 (GPP3) guidelines on communicating industry-sponsored research [2].

**Table 1:** ICMJE recommendations on authorship criteria [1]

Criteria	
1	Substantial contributions to the conception or design of the work; or the acquisition, analysis, or interpretation of data for the work; AND
2	Drafting the work or revising it critically for important intellectual content; AND
3	Final approval of the version to be published; AND
4	Agreement to be accountable for all aspects of the work in ensuring that questions related to the accuracy or integrity of any part of the work are appropriately investigated and resolved.

*ICMJE* International Committee of Medical Journal Editors

These recommendations and guidelines have largely been based on medical publication practices and expectations in North America and Europe, despite an increasing volume of publications in the Asia-Pacific region [3–5]. This increase is likely the result of pharmaceutical companies in the Asia-Pacific region increasingly performing clinical research in the region, as well as developing and executing regional publication plans [3, 6]. However, awareness of the GPP3 recommendations in lower-income countries, including some countries in the Asia-Pacific region, appears to be low [7, 8]. In addition, limited guidance has been provided on pragmatically applying these recommendations in the Asia-Pacific region.

A number of studies suggest that poor publication practices are more prevalent in the Asia-Pacific region compared with other regions, particularly North America and Europe [7–12], although such assertions have been questioned [13, 14]. Several factors may influence adherence to ethical publication practices in the Asia-Pacific region, such as the ubiquitous pressure to publish, unscrupulous providers of editing or publishing services preying on such pressure, language barriers, cultural practices and/or an absence of awareness of global publication standards [6, 8, 10, 14].

An investigation of authorship practices suggested notable differences between the Asia-Pacific region and elsewhere [7]. A lack of consistency in how guidelines are applied and imbalances between the expectations and practices of junior versus senior researchers are common barriers to applying recommended publication practices and are commonly cited as reasons for publication retraction in the Asia-Pacific region [7, 11]. Similarly, while low in number, a higher incidence of retractions due to other issues surrounding publication ethics, such as plagiarism, duplicate publication, fake peer review and breach of copyright, have been reported in Asia compared with other regions [11].

In turn, potential bias against non-English speaking authors during the peer-review process has been reported, independently of scientific quality [15]. While professional medical writing support improves the quality of reporting of clinical trial results [16], researchers in the Asia-Pacific region can be reluctant to acknowledge professional medical writing support because of perceived shame [6]. Consequently, authors of medical and scientific manuscripts in the Asia-Pacific region are often perceived as not adhering to recommended international publication practices. However, this opinion fails to consider the significant barriers to applying Western publication ethics recommendations in the Asia-Pacific context, especially in the absence of region-specific guidance to assist authors in understanding and navigating guidelines issued by international bodies. Although not-for-profit organisations, such as the International Society for Medical Publication Professionals (ISMPP), have held conferences in the region since 2014, there is a need for published guidance that can reach a wider audience.

This manuscript aims to provide a guide for authors and publications professionals in the Asia-Pacific region on applying the GPP3 and ICMJE guidelines when developing medical publications, particularly publications derived from industry-sponsored research. Definitions of key terms are provided in Table 2.

**Table 2:** Definitions of common terms used in this manuscript

Definition	
Authorship agreement	A statement provided to all authors of a publication prior to initiating publication development that explains the rights, roles, responsibilities and expectations of each of party to manuscript development (for example, authors, study sponsors, professional medical writers, translators etc.)
Disclosures	Statements made by authors that provide full context to how their research is being presented, generally describing factors that could potentially be perceived as influencing their interpretation of their research
Encore presentation	Presentation of data at a conference that is similar to data presented at an earlier conference
Gift authorship	Authorship granted to an individual who does not meet the ICMJE authorship criteria as a means of expressing gratitude to an individual or with the expectation of a receiving something of value in return
Guest authorship	Authorship granted to an individual who does not meet the ICMJE authorship criteria often in an attempt to leverage the reputation or standing of the individual, for example, to increase the perceived quality and/or profile of a publication
Honorary authorship	Authorship granted to an individual who does not meet the ICMJE authorship criteria out of respect for that individual
Publications	The full range of formats published in peer-reviewed journals (for example, original research articles, short reports, reviews, or letters to the editor) [2], as well as conference abstracts and presentations
Publication plan	Plans for communicating research, including information such as the timing of submitting publications (both to conferences and peer-reviewed journals), selection of conferences and journals and proposed authors for each publication, among other relevant information
Publication professional	Professional medical writers, publication planners, and publication managers, usually working either in or for companies [2]
Regional publication plan	A publication plan that is developed with the aim of communicating research to an audience within a geographic scope
Stakeholder	Any person or company who has an interest in the publications process, such as an author or study sponsor

*ICMJE* International Committee of Medical Journal Editors

## Applying the Good Publication Practice 3 guidelines in the Asia-Pacific region

The GPP3 guidelines comprise 10 principles, as listed below, ‘to help individuals and organizations maintain ethical and transparent publication practices and comply with legal and regulatory requirements [2]’, particularly in relation to the publication of industry-sponsored research. Given that these guidelines sometimes include ambiguous language that can be challenging to interpret, particularly for speakers of English as a second language, we provide guidance for applying these guidelines in the context of situations that are relevant to authors in the Asia-Pacific region (Table 3).

**Table 3:** Key recommendations for applying the GPP3 principles in the Asia-Pacific region

GPP3 principle [2]	Key recommendations for the Asia-Pacific region
1. *The design and results of all clinical trials should be reported in a complete, accurate, balanced, transparent, and timely manner.*	• Clinical research that is relevant to patient populations in the Asia-Pacific region should be published in a timely manner, ideally in English, to maximise accessibility. • All data from phase 2, 3 and 4 studies should be published in a form that is publicly accessible, regardless of outcome. • To minimise delays in data dissemination; authors should consider making draft manuscripts available via appropriate non-peer-reviewed methods, such as publication via a trial registry, preprint server or a publicly accessible database prior to peer-reviewed publication. • Clinical researchers based in the Asia-Pacific region are encouraged to expedite presentation of their data at regional conferences and to strive for publication in a peer-reviewed journal.
*2. Reporting and publication processes should follow applicable laws (for example, Food and Drug Administration Amendments Act of 2007) and guidelines (for example, ICMJE recommendations and reporting guidelines found on the Enhancing the QUAlity and Transparency Of health Research [EQUATOR] Network).*	• All authors should be aware of any relevant local laws that apply to their research, as well as any laws that may apply to their co-authors and other stakeholders, such as study sponsors. • Translations of key guidelines, such as the ICMJE guidelines, GPP3 and EQUATOR Network checklists should be consulted for clarity, especially by speakers of English as a second language. • Guideline-issuing bodies are encouraged to expedite translations of guidelines into Asian languages to help educate and improve adherence in the Asia-Pacific region.
3. *Journal and congress requirements should be followed, especially ethical guidelines on originality and avoiding redundancy (that is, duplicate publication).*	• Journal and congress requirements should be studied in advance of submitting research for publication. • Encore conference presentations of research previously presented outside of the Asia-Pacific region may be considered for data that are of high regional interest and have not been presented or made readily accessible to local audiences, but care should be taken to ensure that proper approvals and disclosures are made. • The possibility of an encore presentation at a later date should be raised with all authors at the time of preparing the primary publication. • If republishing a translated version of a manuscript, appropriate permissions from the copyright holder (journal and/or authors), journal editors and authors must be sought prior to proceeding, and appropriate efforts made to verify the accuracy of any translation [17, 18].
4. *Publication planning and development should be a collaboration among all persons involved (for example, clinicians, statisticians, researchers, and publication professionals, including medical writers) and reflect the collaborative nature of research and the range of skills required to conduct, analyze, interpret, and report research findings.*	• The publication of Asia-Pacific regional or national data derived from global studies should be planned in advance to limit any delay and ensure timely dissemination of relevant data for patient care in the region. • Collaboration and engagement between stakeholders within and outside the Asia-Pacific region is encouraged to optimise publication outcomes when additional specialist knowledge or skills to support publication development are required.
5. *The rights, roles, requirements, and responsibilities of all contributors (that is, authors and any nonauthor contributors) should be confirmed in writing, ideally at the start of the research and, in all cases, before publication preparation begins.*	• The ICMJE and GPP3 guidelines clearly indicate the expectations of authors of medical and scientific manuscripts published in peer-reviewed journals (as noted earlier, translated versions of these documents are available in Asian languages). • All authors should be made aware of the contribution required to meet all four of the ICMJE criteria before work on a publication begins. • Sponsors or professional medical writers involved in the publication process should provide authorship agreements in a language that will be readily understood by authors. • Potential authors should be identified at the outset of developing a publication and participation from all authors throughout the manuscript development process is strongly recommended. • Authors and study sponsors should consider, develop and proactively communicate processes for managing situations where a proposed author does not meet the ICMJE criteria. • If a prospective author has not met the ICMJE criteria for authorship, the steps necessary to meet ICMJE authorship criteria should be explained and an opportunity provided to fulfil those criteria.
6. *All authors should have access to relevant aggregated study data and other information (for example, the study protocol) required to understand and report research findings.* AND 7. *The authors should take responsibility for the way in which research findings are presented and published, be fully involved at all stages of publication and presentation development, and be willing to take public responsibility for all aspects of the work.*	• All authors should contribute to the writing of a publication for submission to a peer-reviewed journal in accordance with the ICMJE criteria and approve the final version before submission. • Informally approaching individual authors in advance of developing a publication to ensure their understanding of their role and responsibilities (ideally by a speaker of their native language) may be helpful. • An intermediary, such as a medical writer or the study sponsor may assist with collating and incorporating feedback from individuals into a publication to anonymise feedback. • If an author has no comments during the review process, they should clearly communicate that the publication has been thoroughly reviewed before offering no further comment. • Study sponsors and professional medical writers may need to develop novel methods of engaging authors that take into account their preferred ways of working to maximise their contribution, while minimising the review burden, for example, using face-to-face meetings or encouraging junior authors to contribute before approaching senior authors. • Authors should be reminded that by accepting authorship they are jointly responsible for the validity of the research and the integrity/accuracy of the data included in a publication. • Guest, honorary or gift authorship to authors who do not meet the ICMJE criteria must not be permitted. • All authors must provide a ‘Substantial contribution to the conception or design of the work; or the acquisition, analysis, or interpretation of data for the work,’ if performed under their supervision, in addition to critically reviewing and approving the submission of any resulting manuscript. • All individuals who qualify for authorship are named as authors of a manuscript, including employees of study sponsors and junior researchers, when they meet the ICMJE criteria.
8. *Author lists and contributorship statements should accurately reflect all substantial intellectual contributions to the research, data analyses, and publication or presentation development. Relevant contributions from persons who did not qualify as authors should also be disclosed.*	• Defining the scope of the ‘intellectual contribution’ of authors to research may be difficult, but tools are available to guide this decision [19]. • Individual contributorship statements for each author should be made as part of the manuscript and/or cover letter. • Authors should make use of statement templates regarding contributorship statements, and acknowledgment of funding and medical writing/editing provided, by journals or in the GPP3 publication [2]. • Authors should be named at the time of submission. The author list or order should only be revised under exceptional circumstances during the peer-review process.
9. *The role of the sponsor in the design, execution, analysis, reporting, and funding (if applicable) of the research should be fully disclosed in all publications and presentations of the findings. Any involvement by persons or organizations with an interest (financial or nonfinancial) in the findings should also be disclosed.* AND 10. *All authors and contributors should disclose any relationships or potential competing interests relating to the research and its publication or presentation.*	• Transparency regarding any relationships or potential competing interests relating to the research on both an individual and institutional level should always be favoured. • Conflicts of interest including financial, personal, social or other interests that may be perceived to directly or indirectly influence the conduct of the author with respect to manuscript development should be disclosed when submitting a manuscript to a peer-reviewed journal [6, 17]. • The use of translation or English-language editing services and the source of any funding for using such services should be disclosed. • Defaulting to authors having no conflicts of interest is not recommended [20]. • Financial compensation for authoring a publication or presentation is discouraged, although authors may be reimbursed for reasonable publication- or presentation-related out-of-pocket expenses, for example, travel, accommodation and congress registration.

*GPP3* Good Publication Practice 3, *ICMJE* International Committee of Medical Journal Editors

### Requirements for reporting research

1. *The design and results of all clinical trials should be reported in a complete, accurate, balanced, transparent, and timely manner.*

The publication of clinical research, especially research that is specifically relevant to patient populations in the Asia-Pacific region (for example, Asian subgroup analyses) should be published in a timely manner. Publication in English is encouraged, when possible, to maximise accessibility within the region and around the world. English-language fluency should not be a barrier to publication in peer-reviewed journals, and many journals can recommend editing services to help improve writing quality.

All data from phase 2, 3 and 4 studies should be published in a form that is publicly accessible, regardless of the outcome. Phase 3 data should be published within 18 months of the last visit of the last patient. Data can also be made available to the public prior to peer-reviewed publication via appropriate non-peer-reviewed methods, such as publication via a trial registry, preprint server such as bioRxiv, medRxiv or PeerJ preprints, or a publicly accessible database. This can minimise delays in public data dissemination, provide transparency by demonstrating the evolution of a manuscript as author comments are incorporated and improve the quality of the manuscript by soliciting broad feedback from the scientific community prior to, or in parallel with, formal peer review at a journal. This approach should not compromise the ability to submit to a peer-reviewed journal, but the publication policy of any target journal should be checked in advance.

Publication of data generated as part of multinational studies that are relevant to the Asia-Pacific region is often delayed due to subgroup analyses being published after the primary analysis, but any delays should be minimised. Therefore, it is recommended subgroup analyses, including those in Asian populations, be planned in advance to help expedite publication.

In some cases, patient populations in the Asia-Pacific region may be distinct from those assessed in international studies. For example, clinically relevant differences in efficacy, safety and/or pharmacokinetic properties may exist between Western populations and those in the Asia-Pacific region. Under these circumstances, clinical researchers based in the Asia-Pacific region are encouraged to expedite the presentation of their data at regional conferences and to strive for publication in a peer-reviewed journal.

2. *Reporting and publication processes should follow applicable laws (for example, Food and Drug Administration Amendments Act of 2007) and guidelines (for example, ICMJE recommendations and reporting guidelines found on the Enhancing the QUAlity and Transparency Of health Research [EQUATOR] Network).*

All stakeholders should be aware of any relevant local laws that apply to clinical studies and the dissemination of research findings, such as the ‘Korean Sunshine Act’ (Article 47-2 of the Pharmaceutical Affairs Act and Article 13-2 of the Medical Devices Act of Korea), The Philippines Department of Health’s guidelines on pharmaceutical marketing and promotions (Administrative Order N.2015—0053) and Indonesian Sponsorship for Healthcare Professionals: Regulation 58. Relevant local self-regulatory activities, such as the Medicines Australia Code of Conduct and the Japan Pharmaceutical Manufacturers Association’s Transparency Guideline for the Relation between Corporate Activities and Medical Institutions, should also be considered. Stakeholders based in the Asia-Pacific region should also be conscious of, and respect, laws that extend beyond national borders, such as the United States of America (US) Foreign Corrupt Practices Act of 1997, US Sunshine Act (Section 6002 of the Patient Protection and Affordable Care Act [2010]; when working with healthcare professionals based in the USA), General Data Protection Regulations (when handling personal data from citizens of the European Union) and the UK Antibribery Act 2010.

In addition, several key guidelines have been translated into languages that are used in the Asia-Pacific region. For example, the ICMJE guidelines are available in Japanese, Korean, Chinese and Persian, as well as several European languages. Chinese and Japanese translations of the GPP3 guidelines are also available (https://www.ismpp.org/gpp3-translations).

Authors in the Asia-Pacific region are encouraged to consult the EQUATOR guidelines, which provide an effective checklist of key requirements for manuscripts. However, translations into Asian languages can be difficult to find, which represents a potential barrier to non-native English speakers. Furthermore, translations are not universal (for example, CONsolidated Standards Of Reporting Trials [CONSORT] is translated into Japanese, Chinese, Persian and Vietnamese, but not Korean, while Preferred Reporting Items for Systematic reviews and Meta-Analyses [PRISMA] is available in Japanese, Chinese and Korean, but not Persian or Vietnamese). See Case Study 1 in Additional file 1 for details of how applying the EQUATOR Network guidelines when preparing a manuscript can improve outcomes for medical publications.

3. *Journal and congress requirements should be followed, especially ethical guidelines on originality and avoiding redundancy (that is, duplicate publication).*

Journal and congress requirements should be studied in advance of submitting research for publication. Timely access to international or overseas congress data is limited in the Asia-Pacific region. Therefore, encore presentations at regional and national Asia-Pacific events should be considered for data that are of high interest. However, there are several key considerations in doing so.

The possibility of an encore presentation at a later date should be raised with all authors at the time of preparing the primary publication. This may be streamlined by providing a single authorship agreement that relates to the primary publication and any encore publications, subject to the ICMJE authorship criteria (see Table 1). This may include an agreement that additional authors may be added to encore presentations, for example, if the encore must be delivered by an author who is a speaker of a language other than English. The same authorship criteria used for journal publications should be used for congress presentations. Furthermore, any prior presentation should always be acknowledged and a study identifier included as a link between data generated from a common study [21].

Authors and stakeholders should also enquire about whether encore presentations are accepted prior to submitting an abstract to a conference [21]. Copyright of the original conference should also be respected—in some cases, the original conference organiser may require that statements are made acknowledging the original abstract, and that permission has been sought for re-use.

Any encore presentation should also be consistent with the earlier presentation in its scope, but prepared in a manner that is appropriate for the conference and an Asia-Pacific audience [21].

Opportunities may exist to republish in other languages articles initially published in English. If republishing a translated version of a publication, appropriate permissions from the copyright holder (journal and/or authors), journal editors and authors must be sought [17, 18]. Appropriate efforts should also be made to verify the accuracy of any translation and authors are recommended to consult and comply with the guidance provided by the World Association of Medical Editors (WAME) and ICMJE on duplicate publication in another language [17, 18].

### Roles and responsibilities of stakeholders

4. *Publication planning and development should be a collaboration among all persons involved (for example, clinicians, statisticians, researchers, and publication professionals, including medical writers) and reflect the collaborative nature of research and the range of skills required to conduct, analyze, interpret, and report research findings.*

The publication of Asia-Pacific data derived from global studies should be planned in advance to limit any delay in the dissemination of relevant data in the region. These plans should be prospectively communicated and approved by key stakeholders to streamline the publication process. Collaboration and engagement between stakeholders globally, and within the region, is encouraged to optimise publications outcomes. For example, individuals involved with publication planning in the Asia-Pacific region should strive to integrate their plans with those of colleagues in other regions. Collaboration may help expedite publication and provide an external perspective on the value of all data—positive, negative or inconclusive. This also helps avoid duplicate publication. In particular, authors should remember the value of publishing data that are not clearly positive and should not conflate positive study results with a positive reputation.

Examples of how publication professionals in the Asia-Pacific region can play an active role in publication planning are provided in Case Studies 2 and 3 in Additional file 1.

5. *The rights, roles, requirements, and responsibilities of all contributors (that is, authors and any nonauthor contributors) should be confirmed in writing, ideally at the start of the research and, in all cases, before publication preparation begins.*

The ICMJE and GPP3 guidelines clearly indicate the expectations of authors of manuscripts published in peer-reviewed medical journals. Furthermore, the Joint Position Statement from the American Medical Writers Association (AMWA), the European Medical Writers Association (EMWA) and ISMPP, which is available in multiple languages, including Chinese and Japanese, provides an overview of the role of professional medical writers in the development of medical and scientific publications [20].

All authors should be informed that they are required to meet all four of the ICMJE authorship criteria before work on a publication begins (see Table 1). Where collaboration between authors is facilitated by a study sponsor or professional medical writer, it is recommended that an authorship form is provided to each of the potential authors that explains these criteria, ensuring awareness and understanding of the authors’ responsibilities in advance. It may also be useful to provide additional documents explaining the role of each stakeholder, their expectations and the boundaries of their responsibilities. Where a professional medical writer is involved, authors must be asked if they agree to the writer’s involvement before work begins, provide input and approve the general content and direction of the publication through all stages of development. Likewise, there should be a clear differentiation between the roles of an editor, professional medical writer and a translator, as each provides a different service.

Sponsors or professional medical writers involved in the manuscript development process should provide authorship agreements in a language that will be readily understood by each of the authors. Alternatively, the agreement should be written in ‘plain English’ that would be readily understandable for authors who speak English as a second language. Authorship agreements should also be in place for all authors prior to commencing work on a publication. This may also include defining the roles of the lead or first author and corresponding author. An example of details to consider including in an authorship agreement, and why, have been previously published and can be found online (https://www.ismpp.org/assets/docs/Education/AnnualMeeting/5thAM/PosterPresentations/author%20agreement%20forms.pdf) [22].

Potential authors should be identified at the outset of developing a manuscript. Lead authors may wish to avoid defining authorship seniority until the time of submission, once relative contributions to manuscript development are clear, allowing for discussion and mutual agreement. Ideally, consensus should be reached among the authors regarding seniority.

Authors and study sponsors should consider, develop and proactively communicate processes for managing situations where a proposed author does not meet the ICMJE criteria. Setting expectations at the outset may assist in ensuring engagement and contribution from all authors. If an author has not met the criteria to qualify for authorship, then the steps necessary to meet the ICMJE authorship criteria should be reiterated and the prospective author given the opportunity to fulfil those criteria. Alternatively, the prospective author may be offered an acknowledgement.

Stakeholders must also be mindful of competing priorities. For example, in situations where following up with senior authors may not be culturally acceptable, a desire to avoid delays in submitting a manuscript for publication should not override the need for input and approval to submit from every author. It is important to remember that senior authors may wish to offer comment and expert insight prior to submission and that submission is dependent on their approval. However, all stakeholders need to be adaptable to different authors’ methods of working and communication.

A case study on effective multinational collaboration in publication development in the Asia-Pacific region is provided in Case Study 4 in Additional file 1.

### Authorship

6. *All authors should have access to relevant aggregated study data and other information (for example, the study protocol) required to understand and report research findings.*

AND

7. *The authors should take responsibility for the way in which research findings are presented and published, be fully involved at all stages of publication and presentation development, and be willing to take public responsibility for all aspects of the work.*

Explaining the roles and responsibilities of authors prior to developing a publication is essential. If a group discussion among the authors and other stakeholders will be used to formally initiate publication development, informally approaching individual authors in advance to ensure their understanding of their role and responsibilities (ideally by a speaker of their native language) may be helpful.

All authors should contribute to the development of publications and approve the final version before it is submitted, as recommended by the ICMJE. As noted in GPP3 [2] and the Good Practice for Conference Abstracts and Presentations (GPCAP) guidelines [21], a maximum of 10 authors is recommended for an individual publication. Developing a manuscript with contributions from more than 10 authors, while ensuring that agreement is reached on the final content prior to submission, can be challenging to achieve.

In situations where authors are not comfortable providing feedback to their colleagues, an intermediary, such as a professional medical writer or representative of the study sponsor, may assist with collating and incorporating feedback from individuals. Any feedback requiring discussion among all authors can then be anonymised. The lead author may be asked to adjudicate on any conflicting comments. Alternatively, authors can provide consolidated comments representing the views of more than one author as part of the revision process.

If an author has no comments during the review process, they should clearly communicate that they have thoroughly reviewed the manuscript. Some stakeholders may wish to use technology to electronically track the opening and review of documents, but should advise authors in advance if such technology is used.

The scope of ‘drafting the work or revising it critically for important intellectual content’ has not been clearly defined, but proposed definitions of what constitutes a substantial contribution to the development of a manuscript have been published [23]. In the North American and European context, this has been interpreted as authors providing feedback on numerous drafts of a manuscript. However, consideration is needed as to what may be reasonably expected of authors in the Asia-Pacific region. Study sponsors and professional medical writers may need to develop novel methods of engaging authors to maximise their contribution. For example, when delivering a draft publication, a study sponsor or professional medical writer may schedule a face-to-face meeting with an author to allow them to dictate their comments. Alternatively, senior authors in the Asia-Pacific region may prefer to only be asked for comment after their junior collaborators have first provided their input. Whenever possible, authors should be supported through the publication process by a speaker of their native language to ensure understanding of roles and responsibilities and accurate recording of comments.

Authors should be reminded that, by accepting authorship, they are jointly responsible for the validity of the research and the integrity/accuracy of the data included in any publication. Therefore, all authors, regardless of seniority, should have access to all data related to the study. For speakers of English as a second language, care should be taken to ensure that any translation or English-language editing service maintains the integrity of the publication. Academic discussion, led by the lead author or professional medical writer, should be encouraged to resolve any disagreements. If an impasse is reached, an author may wish to consider politely declining authorship.

Guest, honorary or gift authorship to authors who do not meet the ICMJE criteria must not be permitted. While such authorship is commonly offered to Heads of Department and other senior researchers within the Asia-Pacific region, these authors must provide a ‘Substantial contribution to the conception or design of the work; or the acquisition, analysis, or interpretation of data for the work,’ if performed under their supervision, in addition to critically reviewing and approving the submission of any resulting manuscript. The US National Institutes of Health have provided a useful tool for assessing whether a supervisor qualifies for authorship in this regard [19]. Likewise, it has been suggested that performing technical editing, language editing or proofreading; collating author comments; and making minor corrections for grammar, language, formatting or layout does not constitute a substantial contribution to the manuscript [23].

It should be made clear how all authors have contributed to the supervision, conception, analysis and/or interpretation of the research, for example, through contributorship statements in the cover letter and manuscript (see Statement 8). Even if these senior researchers are ultimately responsible for the research nominally performed under their supervision, this, by itself, does not qualify the researcher for authorship. Likewise, all individuals who qualify for authorship should be named as authors of a manuscript, including employees of study sponsors or junior researchers who have met the ICMJE criteria.

Some study sponsors may have policies that require an employee of the sponsor be a named author. The involvement of the study sponsor in the manuscript development process should not be understated. Furthermore, in some instances, a professional medical writer may qualify as an author according to the ICMJE authorship criteria (for example, a review article where the medical writer did the literature research and drafted the article) and should be given authorship.

Instances of authorship being offered for sale, which have been reported in the Asia-Pacific region, are not acceptable under any circumstances [9].

### Transparency

8. *Author lists and contributorship statements should accurately reflect all substantial intellectual contributions to the research, data analyses, and publication or presentation development. Relevant contributions from persons who did not qualify as authors should also be disclosed.*

Defining the scope of the ‘intellectual contribution’ of authors to research, including supervisors, mentors and other potential contributors, may be difficult, although many medical journals require authors to define individual contributions in the manuscript and/or cover letter. Some journals provide examples of authorship statements to guide authors. The US National Institutes of Health has provided a useful pictorial guide of demonstrating what contributions may support a claim to authorship and the strength of such claims [19]. This may be provided to authors to explain the expectations surrounding authorship, particularly if it is adapted and translated to meet local needs. Authors may also be offered a list of potential contributions in their native language to provide a record of their contribution and help draft contributorship statements for publications. The author list should only be revised during the peer-review process under exceptional circumstances.

The GPP3 publication provides statement templates that may be used to disclose funding sources for research, statistical analysis and professional medical writing or editing support [2].

9. *The role of the sponsor in the design, execution, analysis, reporting, and funding (if applicable) of the research should be fully disclosed in all publications and presentations of the findings. Any involvement by persons or organizations with an interest (financial or nonfinancial) in the findings should also be disclosed.*

AND

10. *All authors and contributors should disclose any relationships or potential competing interests relating to the research and its publication or presentation.*

Transparency regarding any relationships or potential competing interests relating to the research on both an individual and institutional level should always be encouraged since disclosing potential or perceived conflicts of interest is unlikely to negatively impact the chance of acceptance for publication [24]. Disclosure merely allows the reader to consider research in the context of the authors’ potentially competing interests.

Conflicts of interest have not been uniformly defined but include financial, personal, social or other interests that may be perceived as directly or indirectly influencing the conduct of the author with respect to manuscript development [7, 24]. Therefore, authors should carefully consider the conflict of interest and disclosure policies of individual journals when submitting their research for publication. In lieu of journal-specific guidance regarding disclosure, the ICMJE conflict of interest disclosure form should be used (http://www.icmje.org/conflicts-of-interest/). Defaulting to authors having no conflicts of interest is not recommended because failing to declare potential conflicts of interest is more likely to result in a negative outcome both during the peer-review process and post-publication compared with making appropriate declarations [20]. Offering authors a tick-box list of common disclosures of potential conflicts of interest in their native language may also help prompt full disclosure.

Many journals do not require acknowledgement of individuals providing English-language editor services or the source of funding for any such support. However, this support should be disclosed, as required for any other professional services used during manuscript development.

Financial compensation for authoring a publication or presentation is discouraged, although authors may be reimbursed for reasonable publication- or presentation-related out-of-pocket expenses, such as travel, accommodation and congress registration expenses.

### Additional considerations

#### Open Researcher and Contributor Identifier (ORCiD) numbers

All authors in the Asia-Pacific region should be encouraged to register for an ORCiD identifier to facilitate clear identification of individual authors. Cross-cultural differences in the use of first versus last names in the Asia-Pacific region versus Europe and North America can make identifying common authors across multiple manuscripts difficult.

#### Data sharing

Data sharing requirements have been introduced by the ICMJE and many journals require data sharing statements to be incorporated into manuscripts [25]. Anecdotally, awareness of data-sharing requirements in the Asia-Pacific region is low. Replicating efforts to communicate data-sharing requirements in medical journals published in Asian languages, as has been done in local Polish [26] and Portuguese [27] journals, may be a first step, in addition to general communication via journal instructions to authors, publications-focused conferences and the ICMJE website.

Guidance on how data will be shared is lacking. Principles for sharing data that are not curated in the English language are an ongoing concern, which may make compliance particularly onerous for researchers in the Asia-Pacific region versus other regions worldwide. As such, it remains unknown how researchers should interpret and manage any data sharing requests, and what potential barriers to data sharing may emerge. Data sharing requirements may also need to be considered as part of the publication planning process.

#### Predatory journals and conferences: a clear and present danger

Institutions and researchers need to be vigilant regarding submitting to, remunerating and recognising the legitimacy of so-called ‘predatory’ or ‘pseudo’ journals [28]. There is currently no generally accepted definition of a predatory journal or conference, but common characteristics are deceptive conduct, a lack of transparency, poor quality standards and unethical publication practices [29].

In general, researchers should be wary of unsolicited communications offering opportunities to publish or suspiciously low publication fees [28, 30, 31]. Publication in a predatory journal may have unforeseen consequences, including reputational damage and its implications for career progression, the inability to publish in a more reputable journal, a lack of discoverability in commonly searched publication databases (for example, MEDLINE, PubMed, EMBASE) and a risk of the manuscript being lost if the journal collapses [28, 32]. If unsure, manual verification of the journal and tools, such as journal selectors (for example, the Directory of Open Access Journals [DOAJ], Journal/Author Name Estimator [JANE]) and thinkchecksubmit.org, are available to help researchers assess their journal choice [28, 33]. Accordingly, medical and research departments in developing Asian countries are encouraged to educate both early career and experienced researchers on methods of avoiding fraudulent journals [33].

### Leadership on publication ethics in the Asia-Pacific region

Journals, medical publication professionals, research institutions and leading researchers in the region need to provide greater leadership regarding ethical publication practices. Several studies of the disclosure requirements for journals in the Asia-Pacific region have illustrated inconsistent practices, with a relatively high proportion of journals having no conflict of interest policy or requirement for disclosures within published manuscripts [34–36].

Efforts to translate relevant guidelines into languages commonly used in the region are encouraged to improve the accessibility, understanding and application of ethical publication practices. To support this, bodies developing guidelines should consider allocating a translation budget to ensure timely production of high-quality translations. For example, readily accessible translations of EQUATOR Network guidelines would represent an important step in advancing data reporting in the Asia-Pacific region.

A level of self-regulation and education is required to achieve improved acceptance and application of ethical publication practices in the Asia-Pacific region, possibly driven by research integrity champions [7]. Some steps have already been taken in this regard, for example, ISMPP has held conferences in China, India, Japan and Singapore to improve publication practices, and the Association for the Promotion of Research Integrity (APRIN) has provided online educational opportunities. Funding organisations, such as the science ministry, the health ministry and National Natural Science Foundation of China (NSFC) in China have also introduced initiatives to combat unethical publication practices [37]. However, many attendees at such meetings are industry stakeholders who are already familiar with the ICMJE and GPP3 guidelines. Therefore, additional effort is needed from local government and regulatory bodies, academic institutions, medical societies and physician associations to promote widespread uptake and application of relevant guidelines on ethical publication practices.

## Conclusions

There is a lack of awareness and understanding of international guidelines on ethical publication practices in the Asia-Pacific region. This commentary provides practical guidance for authors in the Asia-Pacific region to align their publication practices with their international peers and improve the quality of their publications. This is the first tailored practical guidance for the Asia-Pacific region and aims to act as a foundation from which to build improved ethical publication practices in the region.

## Additional file

Additional file 1: Supplementary Appendix (DOCX 22 kb)

## Data Availability

Data sharing is not applicable to this article as no datasets were generated or analysed during the current study.

## Abbreviations

AMWA: American Medical Writers Association
APRIN: Association for the Promotion of Research Integrity
CONSORT: CONsolidated Standards Of Reporting Trials
DOAJ: Directory of Open Access Journals
EMWA: European Medical Writers Association
EQUATOR: Enhancing the QUAlity and Transparency Of health Research
GPCAP: Good Practice for Conference Abstracts and Presentations
GPP3: Good Publication Practice 3
ICMJE: International Committee of Medical Journal Editors
ISMPP: International Society for Medical Publication Professionals
JANE: Journal/Author Name Estimator
NSFC: National Natural Science Foundation of China
ORCiD: Open Researcher and Contributor Identifier
PRISMA: Preferred Reporting Items for Systematic reviews and Meta-Analyses
US: United States of America
WAME: World Association of Medical Editors
