# Supplementary material for: A guide to applying the Good Publication Practice 3 guidelines in the Asia-Pacific region
Source: Res Integr Peer Rev. 2019 Oct 2;4:21. doi: 10.1186/s41073-019-0079-1 (PMC6774224; doi:10.1186/s41073-019-0079-1)
Supplement: Supplementary file 1 — Supplementary Appendix (DOCX 22 kb) [file 41073_2019_79_MOESM1_ESM.docx]

**Examples of sources of guidance on ethical publication practices**

*American Medical Writers Association (AMWA)*

- Code of ethics (https://www.amwa.org/page/code_of_ethics)

*Clarity and Openness in Reporting (CORE)*

- Hamilton S, Bernstein AB, Blakey G, et al. Developing the Clarity and Openness in Reporting: E3-based (CORE) Reference user manual for creation of clinical study reports in the era of clinical trials transparency. Res Integr Peer Rev. 2016;1:4.

*Committee on Publication Ethics (COPE)*

- Code of conduct (https://publicationethics.org/resources/code-conduct)

*Council of Science Editors (CSE)*

- CSE’s white paper on promoting integrity in scientific journal publications (https://www.councilscienceeditors.org/resource-library/editorial-policies/white-paper-on-publication-ethics/)

*Enhancing the QUAlity and Transparency Of health Research (EQUATOR) Network reporting guidelines (http://www.equator-network.org/)*

*International Society of Medical Publications Professionals (ISMPP)*

- AMWA-EMWA-ISMPP Joint Position Statement on the Role of Professional Medical Writers. Medical Writing. 2017;26:7–8.
- Battisti WP, Wager E, Baltzer L, *et al*. Good publication practice for communicating company-sponsored medical research: GPP3. Ann Intern Med. 2015;163:461–464.

*Medical Publishing Insights & Practices (MPIP)*

- Clark J, Gonzalez J, Mansi B, et al. Enhancing transparency and efficiency in reporting industry-sponsored clinical research: Report from the Medical Publishing Insights and Practices Initiative. Int J Clin Pract. 2010;64:1028–1033.
- Chipperfield L, Citrome L, Clark J, et al. Authors’ submission toolkit: A practical guide to getting your research published. Curr Med Res Opin. 2010;26:1967–1982.
- Mansi BA, Clark J, David FS, et al. Ten recommendations for closing the credibility gap in reporting industry-sponsored clinical research: A joint journal and pharmaceutical industry perspective. Mayo Clinic Proc. 2012;87:424–429.
- Marušić A, Hren D, Mansi B, et al. Five-step authorship framework to improve transparency in disclosing contributors to industry-sponsored clinical trial publications. BMC Med. 2014;12:197.
- Lineberry N, Berlin JA, Mansi B, et al. Recommendations to improve adverse event reporting in clinical trial publications: A joint pharmaceutical industry/journal editor perspective. BMJ. 2016;355:i5078.

*World Association of Medical Editors (WAME)*

- Recommendation on publication ethics policies for medical journals. (http://wame.org/recommendations-on-publication-ethics-policies-for-medical-journals)
- World Association of Medical Editors Principles of Transparency and Best Practice in Scholarly Publishing (http://wame.org/principles-of-transparency-and-best-practice-in-scholarly-publishing)
- Statistical Analyses and Methods in the Published Literature [SAMPL] guidelines (http://www.wame.org/Docs/SAMPL-Guidelines-3-13-13.pdf)

**Example case studies of the practical application of the Good Publication Practice 3 recommendations in the Asia-Pacific Region**

**Case study 1**

A company based in the Asia-Pacific region developing a new diagnostic test enlisted the support of a professional medical writer to help develop a manuscript demonstrating the performance characteristics of their test and submit it to a high-impact factor peer reviewed journal. The manuscript underwent peer review, and despite being recommended for publication after addressing the peer reviewers’ minor comments, a peer reviewer recommended that acceptance be conditional on a completed Standards for Reporting Diagnostic accuracy studies (STARD) checklist being submitted. As the medical writer had applied the STARD guidelines when developing the manuscript, no changes were required to the manuscript to meet the STARD requirements and the manuscript was immediately accepted upon presentation of a completed checklist.

This case study illustrates the utility of EQUATOR Network checklists in supporting manuscript development and peer review processes.

**Case study 2**

A publication professional starting a new role in in the Asia-Pacific region found that <5 publications (conference presentations and peer-reviewed manuscripts) were being published each year. The publications professional worked with their colleagues from around the world to identify local knowledge gaps (a topic where knowledge and understanding is lacking or there is a need for increased awareness or understanding) and developed a plan to address these knowledge gaps using scientific publications.

By presenting a publication plan for their local market that was aligned with their global colleagues, the publications professional received an increased resource allocation from the global study sponsor, resulting in the local team increasingd publication output to 30 publications in the first year and 45 the next year, many of which were encore presentations and review articles that targeted local unmet needs and knowledge gaps.

**Case study 3**

A manuscript authors and a regional affiliate lost interest in pursuing a publication after a need for additional data analyses was identified, but insufficient statistical support was available from the parent company. This resulted in delays in data availability, and the publication could not be submitted in time for the local launch of a vaccine that had already received marketing approval in the US and Europe.

To address this situation, a regional medical communications agency invested significant effort in engaging the regional affiliate and authors to revive the manuscript development process, highlighting the need for all stakeholders to play an active role in facilitating the publication of clinical trial data.

**Case study 4**

Cultural norms were making it difficult for a study sponsor to explain to authors what the ICMJE authorship criteria for medical publications are and why they should be followed. This was compounded by professional medical writing support being provided in English by an overseas-based agency.

To address this problem, the study sponsor contracted a local medical writer to support the international team in facilitating conversations with authors during telephone conferences, including discussion surrounding author responsibilities in publication development. Author participation in manuscript development was also facilitated by accepting comments and communicating in Japanese. This process was well received by both the study sponsor, other stakeholders and authors, providing a useful model for engaging authors who do not speak English as a first language about the need to adhere to global publications guidelines.

**Case study 5**

A professional medical writer was asked by an author based in the Asia-Pacific region to ‘ghost write’ a review article and was disappointed when his input was requested. The author was not familiar with the ICMJE authorship criteria and the role of a professional medical writer in supporting manuscript development. This highlights the importance of establishing the roles and responsibilities of all stakeholders before beginning to develop a manuscript, and preferably outlined as part of the authorship agreement. If a professional medical writer is enlisted after manuscript development has started, a teleconference or meeting should be held to ensure all parties are aware of and agree with each other’s roles.

**Case study 6**

A professional medical writer was enlisted by the President of a national medical specialty society in the Asia-Pacific region who had secured financial support from a multinational pharmaceutical company to pay for medical writing and editing services. The medical writer was expected to collaborate with a technical working group comprising society members to adapt recommendations they had formulated for managing a high-burden condition, but did not formally discuss the need to acknowledge financial and medical writing support because both the sponsoring company and the writer at the outset.

After receiving a copy of the final draft, the manuscript was submitted, and accepted for publication by the President, as corresponding author, without informing the sponsoring company or the medical writer. The manuscript also failed to disclose financial or medical writing support.

This case highlights the importance of conducting a formal discussion amongst all stakeholders on the need to appropriately disclose financial and related support for manuscript development so that all parties are aligned on this reporting imperative.
